# Resurrection and typification of *Elatine campylosperma* (Elatinaceae), a long-forgotten waterwort species

**DOI:** 10.7717/peerj.4913

**Published:** 2018-05-29

**Authors:** Attila Takács, Attila Molnár V., Balázs A. Lukács, Timea Nagy, Ádám Lovas-Kiss, Andy J. Green, Agnieszka Popiela, Lajos Somlyay

**Affiliations:** 1 MTA-DE ‘Lendület’ Evolutionary Phylogenomics Research Group, Debrecen, Hungary; 2 Department of Botany, University of Debrecen, Debrecen, Hungary; 3 Department of Tisza Research, MTA Centre for Ecological Research-DRI, Debrecen, Hungary; 4 Department of Plant Sciences and Biotechnology, University of Pannonia, Georgikon Faculty, Keszthely, Hungary; 5 Estación Biológica de Doñana, EBD-CSIC, Seville, Spain; 6 Department of Botany and Nature Conservation, University of Szczecin, Szczecin, Poland; 7 Hungarian Natural History Museum, Budapest, Hungary

**Keywords:** Endozoochory, Amphibious plant, Lectotypification, Herbarium, Mediterranean flora, Wetland ephemerophyte

## Abstract

The name *Elatine campylosperma* Seub. is generally treated as one of the synonyms of *E. macropoda* Guss. However, recent morphological, phylogenetic and karyological studies indicate that this judgement should be revised. In the present paper we typify the name *E. campylosperma*, review its taxonomic history and provide a thorough description, with compilation of previously published data and our new measurements from *in vitro* cultures. Based on our herbarium survey, we outline its Atlantic-Mediterranean distribution area (Spain, France, Italy, Greece, Turkey and Algeria). Habitat preferences are summarized from our field observations, water quality measurements and the label information of the herbarium specimens examined. Intact *E. campylosperma* seeds were found in faecal samples of the Eurasian Coot (*Fulica atra* L.) in southern Spain and two of them were germinated, suggesting that *E. campylosperma* has a capacity for long distance dispersal via endozoochory.

## Introduction

The amphibious genus *Elatine* L. is well-known for its taxonomic complexity, due to the extensive plasticity of their vegetative characters, accompanied by small size, inconspicuous body, ephemeral and clonal life form, poorly known biology and rarity of the included species (Mason, 1956; Coode, 1967; Tucker, 1986; Takács et al., 2013; Molnár et al., 2015). The genus has been the focus of interest for a series of recent studies, addressing distributional (Popiela & Łysko, 2010), ecological (Takács et al., 2013; Minissale & Sciandrello, 2016), morphological (Molnár et al., 2015; Jauzein, 2015; Popiela et al., 2017), phylogenetic (Cai et al., 2016; Sramkó et al., 2016) and evolutionary (Razifard, Les & Tucker, 2017) aspects, which contributed to a more reliable taxonomy of the genus. The severely limited taxonomic relevance of vegetative characters in *Elatine* taxonomy, in contrast to floral and seed traits, was demonstrated by Molnár et al. (2015).

Recent phylogenetic studies (Sramkó et al., 2016; Razifard, Les & Tucker, 2017) confirmed the three main subdivisions of the genus, which were originally established by Seubert (1845) at the rank of sections *Potamopytis* (Adanson) Seub., *Crypta* (Nutt.) Seub., *Elatinella* Seub. The only discrepancy concerned the systematic position of *Elatine brochonii* Clav., for which a separate section had to be created. Focusing on the European representatives of the genus, Sramkó et al. (2016) distinguished two subsections (*Hydropiperia* and *Macropodae*) within sect. *Elatinella*, a controversial group of several, rather poorly known species, the delimitations of which have long been the subject of debate. This is especially true for the subsection *Macropodae*, in which only the type species (*E. macropoda* Guss.) is widely accepted, *E. gussonei* (Sommier) Brullo et al. is only ‘preliminary accepted’ according to Uotila (2009b), whereas *E. campylosperma* Seub. is generally reduced into the synonymy of *E. macropoda* (Uotila, 2009b; see further literature below). Nevertheless, corroborated by the results of Molnár et al. (2015) and Kalinka et al. (2015), Sramkó et al. (2016) accepted the full species status of *E. campylosperma*.

The objectives of the present paper are to: (i) review the taxonomic history of *E. campylosperma*; (ii) typify this name; (iii) provide a thorough description of the morphological traits of *E. campylosperma*, including its diagnostic characters; and (iv) summarize current knowledge of the distribution area and ecology of this species.

## Materials and Methods

The relevant literature on *Elatine* was screened for protologues and further interpretations of the names involved in historical circumscriptions of *E. campylosperma* and related taxa. Historical collections of FI, SASSA, P and TO herbaria were screened for taxonomically and nomenclaturally relevant specimens of the species.

Seeds from indigenous populations of plants with long flower pedicels and strongly curved seeds, which correspond to the description of *E. campylosperma* provided by Moris (1837) and Seubert (1842), were collected from Italy (Sardinia, Giara di Gesturi, 27 April 2012, N 39.739°, E 8.995°) and Spain (Doñana, Marisma del Rocío, 21 April 2013, N 37.128°, W 6.488°) under permit (2015107300000771/FQH/MDCG/mes).

To provide a description, six morphological traits of *E. campylosperma* were investigated and measured on specimens from *in vitro* cultures, following the standard of Molnár et al. (2015). Seeds were sown in plastic boxes on sterilized (autoclaved) soil, which was permanently wetted. Plantlets were grown in climate controlled rooms (with 14 h/day light and 30 μmol m^−2^ s^−1^ light intensity, and temperatures of 22 ± 2 °C under light and 18 ± 2 °C under darkness) until they reached the fruiting stage. A total of six vegetative characters (length of stem, length of internode, length of lamina, width of lamina, length of petioles, length of pedicel) were measured on 50–50 fruiting stems using calipers (0.1 mm accuracy). The numerical variables measured, together with those presented by Glück (1911), were incorporated into the description of the species. Mature fruits were gathered and seed numbers per capsules were counted. Then seeds were pooled and 3 × 100 seeds were measured for the weight of thousand seeds. Curvature of seeds is given after Popiela et al. (2017), and chromosome numbers after Kalinka et al. (2015). Scanning electron microscope (SEM) images of the seeds were taken at ×200 magnification using a SEM (Zeiss Evo) by Magdalena Bihun and Bożenne Białecka (Molecular Biology and Biotechnology Center, University of Szczecin, Szczecin, Poland). Chromosome photographs were taken by Anna Kalinka (Molecular Biology and Biotechnology Center, University of Szczecin, Szczecin, Poland) using the epifluorescence microscope Axio Imager Z2 (Carl Zeiss, Oberkochen, Germany).

Plastid sequences (accD-psaI, psbJ-petA, ycf6-psbM-trnD) produced in a previous study (Sramkó et al., 2016) and deposited in GenBank were aligned and polymorphic sites were assorted by eye.

Specimens of tetramer-flowered, opposite-leaved taxa of *Elatine* (essentially the members of subsect. *Hydropiperia* and *Macropodae*) preserved at B, BP, CL, DE, H, LY, MA, PR, PRC, SEV, TO, UNEX and W herbaria (altogether 293 specimens) were examined and revised to clarify the distribution of *E. campylosperma*. A distribution map was compiled using Quantum GIS 2.18 (Quantum GIS Development Team, 2017) software environment.

Data on the habitat preference of *E. campylosperma* come from field observations (at the sampled localities) as well as label information for the herbarium specimens examined. To characterize habitat salinity, we measured conductivity and pH on the sites using a Hach HQ40D handheld multi meter under permit (2014/30).

Information on the seed dispersal of the species was based on our field observation detailed below, and the following lab work. On 18 March 2016 we observed a flock of >200 Eurasian Coot (*Fulica atra* L.) feeding on an extensive carpet of *E. campylosperma* that was largely above the waterline (Marisma del Rocío, Spain, 37.12503° N, 06.49117° W). We collected 41 fresh faecal samples (under permit 2014/31) deposited by these birds close to the water’s edge, with the aim of looking for seeds of *E. campylosperma* that had survived passage through the digestive system of *F. atra*. The fresh mass of the collected faecal samples was 1.57 g (mean, range: 0.664 g, − 3.85 g). Each sample was placed in an individual plastic zip bag and carefully inspected in the laboratory to remove any material stuck on the outside; they were then stored at 5 °C until processing. For the separation of seeds, we used a 100 μm sieve and deionized water. Each washed sample was inspected under stereomicroscope and plant seeds were separated. Germination tests of intact seeds were conducted in Petri-dishes, on 1% agarose gel, using a 14 h of photoperiod (30 μmol/m^2^/sec light intensity) with a 22 ± 2 °C daytime and 18 ± 2 °C night-time temperature. This initial germination test lasted one month. After that the seeds were stored for one year at a temperature of 4 °C, which was followed by a second germinability test on sterilized (autoclaved for 3 h, 180 °C) soil, which was continuously moistened.

## Results and Discussion

### Taxonomic history of *E. campylosperma*

Since the name *E. campylosperma* has usually been synonymized with, or treated as an infraspecific taxon of, either *E. hydropiper* L. or *E. macropoda*, it is worth briefly reviewing the taxonomic history of the most relevant taxa.

The first species of waterworts characterized by opposite leaves was described by Linnaeus (1753: 367), and named as *E. hydropiper*. The original concept of this species included both tetramerous and trimerous flowered taxa, corresponding to Vaillant’s (1727) ‘*Alsinastrum serpillifolium*, *flore albo tetrapetalo*’ and ‘*Alsinastrum serpillifolium*, *flore roseo tripetalo*’, respectively. The latter taxon was later separated by Schkuhr (1791: 345) as a new species, *E. triandra*. Schkuhr provided accurate pictures of *E. hydropiper* and *E. triandra* (Tab. CIX. b), showing the flower (diplostemonous, tetramerous vs. haplostemonous, trimerous) and seed (considerably curved vs. slightly curved) characteristics of both species. More than 200 years later the name *E. hydropiper* was lectotypified in this sense by Jonsell & Jarvis (2002). Additionally, Schkuhr provided another figure of ‘*E. hydropiper*’ (Tab. CIX., bottom, right-hand one) which he copied from Vaillant’s work (Vaillant, 1727, Tab II. Fig. 2.).

Braun (1824) followed in the footsteps of Vaillant (1727) and Schkuhr (1791). Braun basically accepted Schkuhr’s treatment, but claimed that in Schkuhr (1791) the top picture of *E. hydropiper* on Tab. CIX. b is obviously different from the other picture of the same species on Tab. CIX. (bottom, right-hand one), and that consequently they represent two taxa. Braun explained that the former picture portrays a small plant with relatively long leaves and *sessile* flowers, representing typical *E. hydropiper*, while the latter portrays a robust plant with shorter and petiolated leaves as well as *pedicellate* (‘*pedunculate*’) flowers. Although Braun, as well as Schkuhr, admittedly had not seen the latter taxon in nature, and hesitated over whether it was a plain variety of *E. hydropiper*, finally he decided to describe it as a new species, *E. major*. Unfortunately, in the protologue (Braun, 1824) nothing was said about the shape of seeds, which is nowadays considered one of the taxonomically most valuable characters in this genus (Molnár et al., 2015; Popiela et al., 2017). Although the name *E. major* is generally considered a plain synonym of *E. hydropiper* (Kerguélen, 1999; Uotila, 2009a; Popiela et al., 2012), it has actually been unclear for almost two centuries to which pedicellate and tetramerous flowered *Elatine* species Braun’s name should be assigned. The slightly curved, almost straight seed shape of *E. major* has recently been described by Jauzein (2015), on the basis of plants from the *locus classicus* of this taxon, i.e. the locality from where Vaillant (1727: 5) reported his ‘*Alsinastrum serpillifolium, flore albo tetrapetalo*’ (Fontainebleau forest, France). From a unique combination of plant characters, Jauzein (2015) postulated the endemic status of the Fontainebleau plant. This remains to be seen, however, because *E. major* has not yet been involved in comparative morphological and molecular research. Importantly for our study, on the basis of its seed shape and short pedicel this taxon is obviously distinct from *E. campylosperma*.

Gussone (1827) described another pedicellate and tetramerous flowered species, *E. macropoda* from Sicily. Unfortunately, no morphological comparison with Braun’s *E. major* was made, and the shape of seed was not described in the protologue. Although Gussone referred to a picture of his species in ‘Fl. sic. t. 204. f. 1.’, this reference remains an unsolved mystery until the present day. Most probably, the referred illustration has never been published (D. Iamonico, G. Domina and A. Santangelo, 2013, personal communication). As is clear from the synopsis of Gussone (1842: 458), he was uncertain about the typical seed shape of his *E. macropoda*, and attributed the almost straight seeds of his own specimen (stored in NAP herbarium) to their putatively unripe state (‘*quia forsan immatura vix curvata sunt*’). Apparently, Gussone’s (1842) speculation was driven by the treatment of Bertoloni (1839), who characterized *E. macropoda* as a species with highly curved seeds (‘*seminibus exquisite curvatis*’). In fact, Bertoloni unsuccessfully combined Gussone’s *E. macropoda* (with slightly curved or almost straight seeds) with Moris’s (1837) ‘*E. hydropiper pedunculata*’ (with highly curved seeds; see below), thus matching the name *E. macropoda* with the seed characters of Moris’s taxon. Actually, the slightly curved seeds observed by Gussone on his own specimen are generally indicative of *E. macropoda* (Seubert, 1845; Cook, 1968; Pignatti, 1982; Popiela & Łysko, 2010; Popiela et al., 2017).

The first comprehensive accounts of the genus *Elatine* were implemented by Seubert (1842, 1845), who provided an infrageneric classification of the genus, basically still followed today. Seubert recognized the taxonomic significance of seed shape, and presented elaborate illustrations of most species he accepted (Seubert, 1845). He described a new tetramerous flowered species, *E. campylosperma* (Seubert, 1842: 284), distinguishing it by its long pedicellate flowers (‘*pedunculo folium superante*’) and semicircular seeds (‘*seminibus in semicirculum involutis*’), but failed to illustrate it, even in his monographia (Seubert, 1845). Unfortunately, Seubert cited no specimens in the protologue. He only referred to the description and schematic drawing of ‘*Elatine hydropiper pedunculata*’ that had been described by Moris from Sardinia (‘*In udis maritimis*’, Moris, 1837: 287, Tab. XX. ic. 2). Indeed, Moris characterized his taxon by very long pedicels (‘*plerisque folio valde longioribus*’) and horseshoe-like seeds (‘*seminibus instar ferri equini omnibus constanterque convolutis*’), reliably pictured on the drawing (Tab. XX. ic. 2). This illustration is part of the original material of the name *E. campylosperma* (Art. 9.3. of the ICN McNeill et al., 2012). Accordingly, Seubert (1842) specified the provenance of *E. campylosperma* as ‘*Crescit in udis maritimis Sardiniae*’.

Despite Seubert’s taxonomically reliable works, the species status of *E. campylosperma* was not accepted by the great majority of later authors (Rouy & Foucaud, 1896; Coode, 1967; Cook, 1968; Cirujano & Velayos, 1993; Kerguélen, 1999; Uotila, 2009b; Popiela & Łysko, 2010; Jauzein, 2015), even in Italy (Pignatti, 1982; Bocchieri & Mulas, 1992; Conti et al., 2005; Desfayes, 2008; Bagella et al., 2009; Bagella & Caria, 2012). Among the few exceptions were Moesz (1908), who described *E. hungarica*, distinguishing it from Seubert’s *E. campylosperma*, and Glück (1911), who splitted the latter species into two varieties. Glück’s *E. campylosperma* var. *parviflora* is most probably identical with *E. gussonei*, whereas *E. campylosperma* var. *grandiflora* is fully identical with Seubert’s *E. campylosperma* (see below).

**Figure 1:**
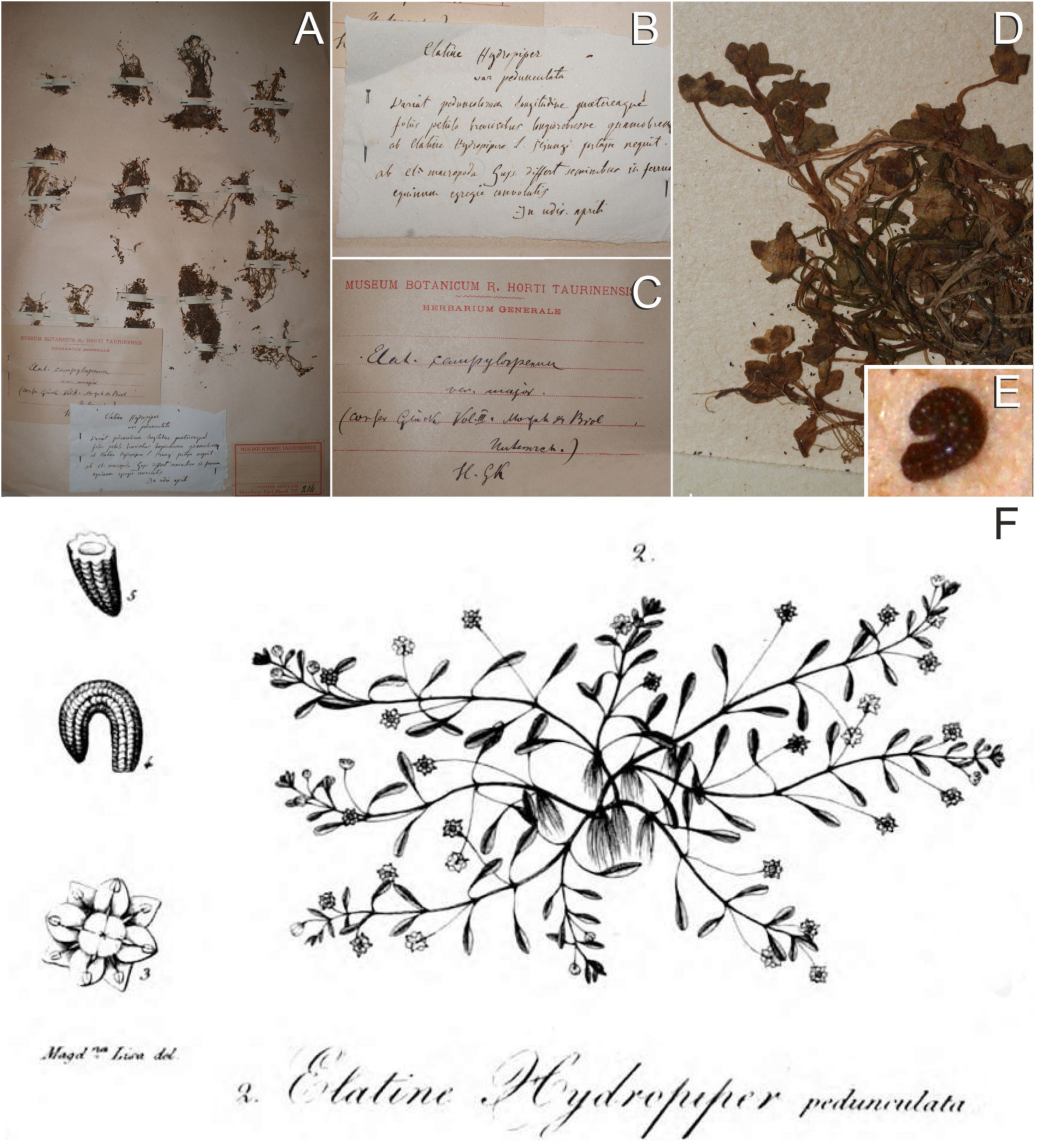
Original material of *E. campylosperma*. (A) Herbarium sheet of *E. hydropiper* var. *pedunculata* (TO, Herb. Moris no. 214.); (B) Label from Moris; (C) Label from Glück; (D) A fragment of the specimen showing long pedicellate flowers; (E) A seed of the specimen; (F) Drawing of *E. hydropiper pedunculata* (Moris, 1837: Tab. XX. ic. 2.). Photographs: (A–E): A. Molnár V.

We had the opportunity to investigate Moris’ major collection in TO herbarium, and traced a single specimen of his ‘*Elatine hydropiper pedunculata*’ (Figs. 1A–1E), unfortunately without clear indication of its provenance and collecting date (‘*In udis. aprili*’). This specimen was catalogued in Barbey’s compendium (Barbey, 1884: 25) under no. 214., which is indicated on a separate label on the specimen. There is another handwritten note attached to the sheet, from Hugo Glück, reading ‘*Elat[ine] campylosperma var. major (confer Glück Vol. III. Morphol[ogische] & Biol[ogische] Untersuch[ungen]) H. Gk.*’. Although Glück used the nickname ‘var. *major*’, his self-citation refers to the description of *E. campylosperma* var. *grandiflora* in Glück (1911). No relevant materials were found in FI and SASSA herbaria (Ch. Nepi and S. Bagella, 2013, personal communication) where Moris may have sent duplicates (Steinberg, 1977; Arrigoni, 2006). However, we have traced a specimen in P herbarium (P05571614) which was collected by Moris in Sardinia, seemingly in 1837, i.e. in the year of publication of his *Flora Sardoa* (Moris, 1837) (image at goo.gl/zcYtNS).

Although the traced specimens (TO, P) may belong to the original material of the name *E. campylosperma*, there are some uncertainties about their status. Firstly, Moris (1837) was not sure that his taxon was distinct from Gussone’s *E. macropoda* (‘*Huccine Elatine macropoda Guss. Fl. sic. Prod. I. p. 475.?*’). On the TO specimen, however, Moris’ hand-written note clearly explains the diagnostic difference in the seed shape between the two species (‘*ab el. macropoda Guss. differt seminibus in ferrum equinum egregie convolutis*’). This discrepancy can be eliminated if we assume that Moris collected the specimen prior to 1837, but added the note subsequently. Secondly, the protologue of Moris’ taxon was published in late April of 1837 (Stafleu & Cowan, 1981), hence it is unlikely that the name ‘*Elatine hydropiper pedunculata*’ was based on the P specimen collected in the same year (if the number ‘1837’ on the label refers to the collecting date at all, and not the year of publication of Flora Sardoa). It is worth mentioning that Moris had already recorded ‘*E. hydropiper*’ in Sardinia in the early years of his field researches (Moris, 1827: 7).

Nonetheless, Moris’ (1837) drawing (Fig. 1F) unequivocally belongs to the original material, and permits a precise application of the name, therefore this illustration is designated here as the lectotype of the name *E. campylosperma*.

### Taxonomic treatment

***Elatine campylosperma*** Seub. in Walpers, *Repert. Bot. Syst*. 1: 284. 1842.—Lectotype (designated here by Somlyay): [icon] ‘*Elatine hydropiper pedunculata*’ in Moris, *Fl. Sardoa* 1: Tab. XX. ic. 2. 1837. ≡ *Potamopitys campylosperma* (Seub.) Kuntze, *Revis. Gen. Pl*. 1: 58. 1891.

= *E. campylosperma* var. *grandiflora* Glück, *Biol. Morphol. Untersuch. Wasser-Sumpfgewächse* 3: 318. 1911. Type: not designated.

Etymology—The species epithet is combined from the Greek word ‘*kampylos*’ (καμπυλoς) (= curved) and the Latin word ‘*sperma*’ (= seed), which refers to the characteristic seed shape of the species.

Illustrations—Moris (1837): Tab. XX. ic. 2. (habit, flower, seed); Glück (1911): Fig. 28. A–B. (flower, fruit); Sramkó et al. (2016): Fig. 1. I. (habit, flower); Popiela et al. (2017): Fig. 8 G–I. (seed coat structure), Fig. 10 A–E. (seed).

**Figure 2:**
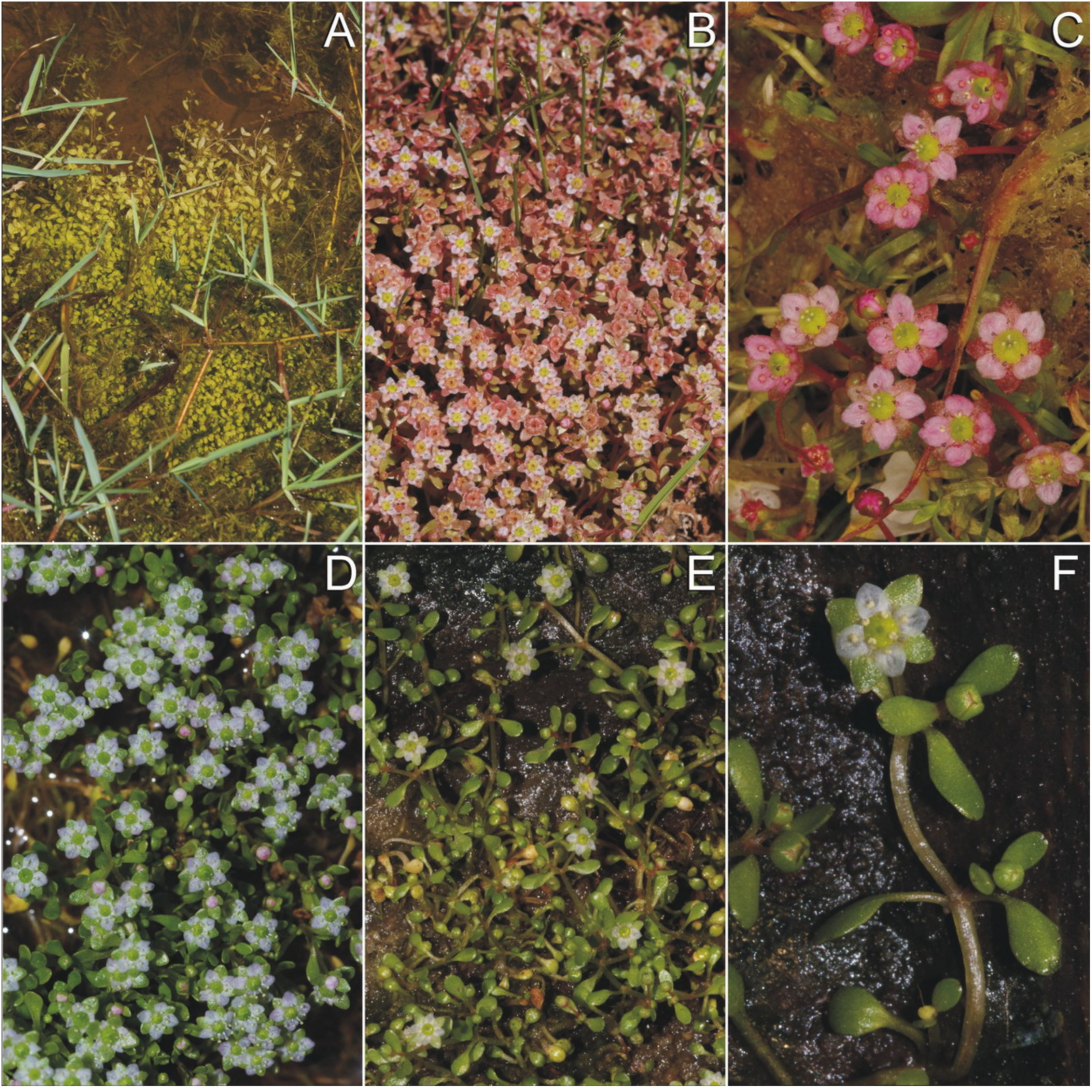
Habit of *E. campylosperma*. (A) Aquatic form (Sp: El Rocío); (B) Flowering and fruiting specimens in full sunlight (Sp: El Rocío); (C) Flowering specimens with intensive pink petals (It: Giara di Gesturi); (D) Cultivated plants (originated from It: Giara di Gesturi); (E and F). Cultivated plants (originated from Sp: El Rocío). Photographs: (A–B) and (D–F): *A. Molnár V.*, (C): *B. A. Lukács.*

Description—Annual plant, typically with extensive clonal patches (Figs. 2A and 2B). The procumbent or emergent stems are (4–)11–22(–36) mm in length. The lamina is (1.3–)2–3(–13) mm in length and (0.7–)1–2(–2.6) in width, with a petiole of (0.5–)1–3.5(–10) mm, cuneate at the base and rounded at the apex, standing in an opposite position. Internodes are (1–)3–7(–13) mm in length. The tetramerous flowers have long pedicels (see below), arising one by one from the leaf armpits. There are four stamens and the stigma is four-lobed. Sepals are ovate, and widest at the base. Petals are ovate, as long as the sepals, bluntly acute at the tip (Figs. 1F and 2B–2F), white (Figs. 2D–2F) or —depending on the light conditions — pink (Figs. 2B and 2C). Under bright sunlight, the whole shoot changes to pink (Fig. 2B). The capsules are globose or slightly depressed, divided to four equal compartments. Number of seeds per capsules is (1–)3–12(–20). Seeds are strongly curved (see below). The thousand-seed weight was 0.0194 g (Giara di Gesturi) and 0.0116 g (El Rocío). No cleistogamous flowers were observed, neither on indigenous plants nor on the *in vitro* cultures.

**Figure 3:**
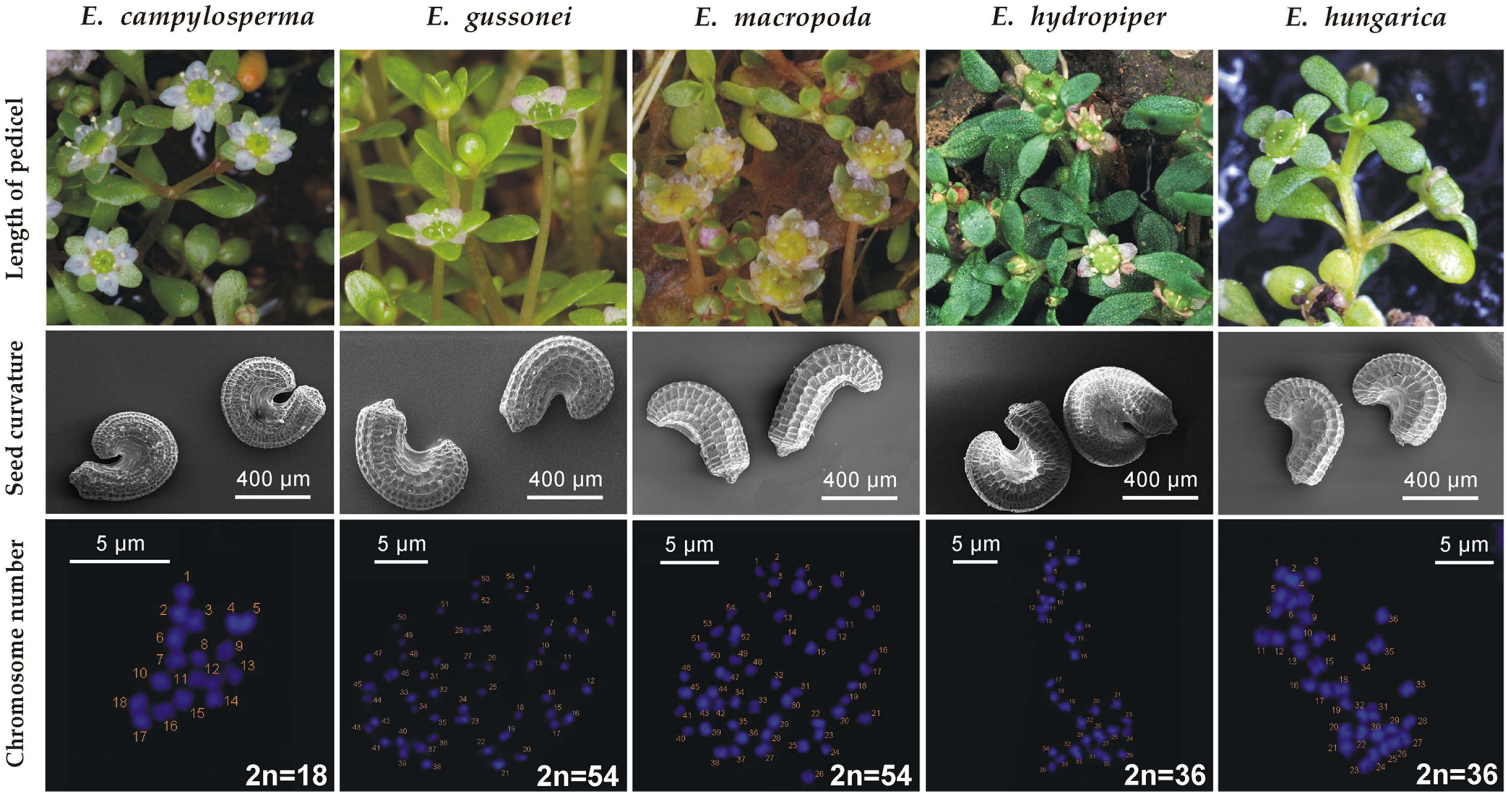
Comparison of *E. campylosperma* and the most similar species. Origin of the specimens: *E. campylosperma*: It: Sardinia, Giara di Gesturi; *E. gussonei*: It: Lampedusa; *E. macropoda*: It: Sardinia, Olmedo; *E. hydropiper*: Hu: Tiszagyenda; *E. hungarica*: Hu: Konyár. Photographs: flowering shoots: *A. Molnár V*.; seeds: *B. Białecka* & *M. Bihun*; chromosomes: *A. Kalinka*.

Diagnostic characters—Corresponding to Moris (1837) and Seubert (1842), the long flower pedicel ([1–]3–7[–10,5] mm) distinguishes it from *E. hydropiper*, which has almost sessile flowers, both in its aquatic and terrestrial forms. The strongly curved ([222–]265–294[–337]°) seeds distinguishes it from *E. macropoda* ([78–]111–134[–167]°), *E. gussonei* ([80–]180–247[–347]°) and *E. hungarica* ([161–]213–247[–299]°) (Popiela et al., 2017) (furthermore, *E. hydropiper* and *E. hungarica* have different geographical distributions). The seed coat reticulation of *E. campylosperma* is composed of (15–)31–42(–59) narrow rectangular pits in the middle row, whereas *E. macropoda* has (13–)19–23(–29) rectangular pits, and *E. gussonei* and *E. hungarica* have 17–23(–32) and (11–)20–26(–35) hexagonal pits in the same position respectively (Popiela et al., 2017). The main diagnostic differences between *E. campylosperma* and the most similar taxa are visualized in Fig. 3.

Plastid sequences produced in a previous study (Sramkó et al., 2016) show several informative sites where *E. campylosperma* consistently differs from other taxa of the subsect. *Hydropiperia* and *Macropodae* (18 sites in accD-psaI, 15 in psbJ-petA, and 14 in ycf6-psbM-trnD intergeneric spacer region, including length polymorphism and base substitutions; Tables 1–3). As was already discussed in Kalinka et al. (2015), *E. campylosperma* is the only diploid plant (2*n* = 18) in the genus known so far.

**Table 1:** Polymorphic sites in accD-psaI intergeneric spacer of 5 *Elatine* taxa, where the motifs are diagnostic for *E. campylosperma*.

Taxa (sample origin)	GenBank accession numbers	0	0	0	0	0	0	0	0	0	0	0	0	0	0	0	0	0	1
		0	4	4	5	5	7	7	7	7	8	8	8	8	8	8	8	9	1
		7	4	2	7	9	1	7	9	9	0	0	0	0	0	3	3	5	3
		0	0	2	6	9	8	3	8	9	0	1	2	3	4	3	4	9	9
*E. campylosperma* (IT)	KX818160	T	C	C	G	A	G	G	T	T	T	T	A	T	T	A	T	C	C
*E. campylosperma* (SP)	KX818161	T	C	C	G	A	G	G	T	T	T	T	A	T	T	A	T	C	C
*E. macropoda* (TR)	KX818166	G	A	A	C	.	T	A	.	.	.	.	.	.	.	C	G	A	A
*E. macropoda* (SP)	KX818165	G	A	A	C	.	T	A	.	.	.	.	.	.	.	C	G	A	A
*E. macropoda* (IT)	KX818167	G	A	A	C	.	T	A	.	.	.	.	.	.	.	C	G	A	A
*E. gussonei* (LMP)	KX818169	G	A	A	C	.	T	A	.	.	.	.	.	.	.	C	G	A	A
*E. gussonei* (SP)	KX818168	G	A	A	C	.	T	A	.	.	.	.	.	.	.	C	G	A	A
*E. gussonei* (MA)	KX818163	G	A	A	C	.	T	A	.	.	.	.	.	.	.	C	G	A	A
*E. gussonei* (MT)	KX818164	G	A	A	C	.	T	A	.	.	.	.	.	.	.	C	G	A	A
*E. gussonei* (IT)	KX818162	G	A	A	C	.	T	A	.	.	.	.	.	.	.	C	G	A	A
*E. hungarica* (HU)	KX818155	G	A	A	C	.	T	A	.	.	.	.	.	.	.	C	G	A	A
*E. hungarica* (RU)	KX818156	G	A	A	C	.	T	A	.	.	.	.	.	.	.	C	G	A	A
*E. hydropiper* (HU)	KX818157	G	A	A	C	.	T	A	.	.	.	.	.	.	.	C	G	A	A
*E. hydropiper* (PL)	KX818158	G	A	A	C	.	T	A	.	.	.	.	.	.	.	C	G	A	A

**Table 2:** Polymorphic sites in psbJ-petA intergeneric spacer of 5 *Elatine* taxa, where the motifs are diagnostic for *E. campylosperma*.

Taxa (sample origin)	GenBank accession numbers	0	0	1	1	1	1	1	1	1	1	1	2	3	4	4
		5	5	4	4	4	5	5	5	5	5	5	4	0	6	7
		6	8	7	8	9	1	2	3	4	5	6	5	5	2	3
*E. campylosperma* (IT)	KX818187	T	G	T	A	C	G	C	A	T	T	T	.	T	T	.
*E. campylosperma* (SP)	KX818188	T	G	T	A	C	G	C	A	T	T	T	.	T	T	.
*E. macropoda* (TR)	KX818193	A	.	.	T	A	.	.	.	.	.	.	T	C	G	T
*E. macropoda* (SP)	KX818192	A	.	A	T	A	.	.	.	.	.	.	T	C	G	T
*E. macropoda* (IT)	KX818194	A	.	A	T	A	.	.	.	.	.	.	T	C	G	T
*E. gussonei* (LMP)	KX818196	A	.	.	T	A	.	.	.	.	.	.	T	C	G	T
*E. gussonei* (SP)	KX818195	A	.	.	T	A	.	.	.	.	.	.	T	C	G	T
*E. gussonei* (MA)	KX818190	A	.	.	T	A	.	.	.	.	.	.	T	C	G	G
*E. gussonei* (MT)	KX818191	A	.	.	T	A	.	.	.	.	.	.	T	C	G	G
*E. gussonei* (IT)	KX818189	A	.	.	T	A	.	.	.	.	.	.	T	C	G	G
*E. hungarica* (HU)	KX818182	A	.	.	T	A	.	.	.	.	.	.	T	C	G	G
*E. hungarica* (RU)	KX818183	A	.	.	T	A	.	.	.	.	.	.	T	C	G	G
*E. hydropiper* (HU)	KX818184	A	.	.	T	A	.	.	.	.	.	.	T	C	G	G
*E. hydropiper* (PL)	KX818185	A	.	.	T	A	.	.	.	.	.	.	T	C	G	G

**Table 3:** Polymorphic sites in ycf6-psbM-trnD intergeneric spacer of 5 *Elatine* taxa, where the motifs are diagnostic for *E. campylosperma*.

Taxa (sample origin)	GenBank accession numbers	0	0	0	0	0	0	0	0	0	0	1	1	1	1
		1	1	2	3	4	5	7	8	8	9	2	3	3	4
		1	4	6	9	5	1	9	0	5	0	9	2	6	6
		4	6	2	4	1	0	1	0	8	1	7	0	2	7
*E. campylosperma* (IT)	KX818133	A	T	A	A	T	A	A	C	A	T	C	G	A	A
*E. campylosperma* (SP)	KX818134	A	T	A	A	T	A	A	C	A	T	C	G	A	A
*E. macropoda* (TR)	KX818139	T	A	C	G	G	T	T	.	C	A	T	T	G	G
*E. macropoda* (SP)	KX818138	T	A	C	G	G	T	T	.	C	A	T	T	G	G
*E. macropoda* (IT)	KX818140	T	A	C	G	G	T	T	.	C	A	T	T	G	G
*E. gussonei* (LMP)	KX818142	.	A	C	G	G	.	T	.	C	A	T	T	G	G
*E. gussonei* (SP)	KX818141	.	A	C	G	G	.	T	.	C	A	T	T	G	G
*E. gussonei* (MA)	KX818136	T	A	C	G	G	T	T	.	C	A	T	T	G	G
*E. gussonei* (MT)	KX818137	T	A	C	G	G	T	T	.	C	A	T	T	G	G
*E. gussonei* (IT)	KX818135	T	A	C	G	G	T	T	.	C	A	T	T	G	G
*E. hungarica* (HU)	KX818128	T	A	C	G	G	T	T	.	C	A	T	T	G	G
*E. hungarica* (RU)	KX818129	T	A	C	G	G	T	T	.	C	A	T	T	G	G
*E. hydropiper* (HU)	KX818130	T	A	C	G	G	T	T	.	C	A	T	T	G	G
*E. hydropiper* (PL)	KX818131	T	A	C	G	G	T	T	.	C	A	T	T	G	G

Key to the representatives of *Elatine* sect. *Elatinella* subsect. *Hydropiperia* and *Macropodae* in Europe:

1a Flowers (almost) sessile, pedicels less than 1 mm long.2
1b Flower pedicels more than 1 mm long.3
2a Seeds strongly curved: (246–)273–291(–318)°.—Seed coat reticulation composed of (22–)37–48(–62) narrow pits in the middle row. Tetraploid (2*n* = 36).*E. hydropiper* L.
2b Seeds slightly curved or almost straight: (55–)61–99(–156)°.—Seed coat reticulation composed of (23–)32–38(–47) narrow pits in the middle row. Tetraploid (2*n* = 36).*E. orthosperma* Düben
3a Seeds slightly curved: (78–)111–134(–167)°.—Seed coat reticulation composed of (13–)19–23(–29) broad pits in the middle row. Hexaploid (2*n* = 54).*E. macropoda* Guss.
3b Seeds strongly curved: ≥200° on average.4
4a Seed coat reticulation composed of narrow pits. Number of pits in the middle row usually more than 30. Seeds extremely curved [(222–)265–294(–337)°]. Diploid (2*n* = 18)*E. campylosperma* Seub.
4b Seed coat reticulation composed of broad pits. Number of pits in the middle row usually less than 30. Seeds relatively less curved.5
5a Length of seeds ≤600 μm, width of seeds ≤400 μm.—Number of pits in the middle row (11–)20–26(–35). Seeds curved in (161–)213–247(–299)°. Tetraploid (2*n* = 36).*E. hungarica* Moesz
5b Length of seeds >600 μm, width of seeds >400 μm.—Number of pits in the middle row 17–23(–32). Seeds curved in (80–)180–247(–347)°. Hexaploid (2*n* = 54).*E. gussonei* Brullo et al.

### Herbarium survey

During the herbarium revision *ca*. 20% of specimens could not be reliably identified to species because of the lack of particular organs (seeds and flowers or fruits). Although the reviewed herbaria contained numerous specimens which had been named as *E. campylosperma*, only approximately one third of them were identified correctly (Table 4). Altogether, 32 specimens were revised or confirmed as *E. campylosperma*. These are as follows: **Algeria**: Bonae, s.d. (<1900), *A. Steinheil* (W, as *E. hydropiper*). **France**: Charente-Inférieure, Marais de Bords, 15 June 1884, *J. Foucaud* (LY, PRC, W). Charente-Inférieure. Bords, mourois. June 1884, *J. Foucaud* (LY). Bords (Ch. Inf.) May 1884, *J. Foucaud* (LY). Environs de Rochefort (Ch.-Inf.). Prairie de Rhosne, 17 August 1886, 24 August 1888, *J. Foucaud* (LY). Marais de Genouillé (Ch.-Inf.), 1875, *J. Foucaud* (LY). St-Urbain. Vendée, July 1854, *Ch. Pontarlier* (BP, W). St-Urbain (Vendée), 23 July 1853, *Ch. Pontarlier* (LY). **Greece**: am Weg von Kalogria nach Loutra Kounoupelli, 14 May 1995, *anonym*, (B). **Italy**: Cerdena. Cagliari. Gesturi. Cercanías del centro didáctico, alcornocal con mirto y lagunas temporales en tabla basáltica, 07 June 2003, *M. Angel Garcia et al.* (MA, as *E. macropoda*). Sardegna, Terralba, Giara di Gesturi, 27 April 2012, *Takács et al.* (DE). Sardinia, s.d. (<1900), *J. H. Moris* (TO, as *E. hydropiper pedunculata*). **Spain**: El Rocio, Huelva, 21 April 2013, *Molnár V. A. et al.* (DE). Huelva: El Rocío. Coto Doñana, 21 May 1970, *P. Gibbs* & *S. Silvestre* (MA, as *E. macropoda*). Huelva: Hinojos Marismas, April 1978, *S. Talavera* (MA, as *E. macropoda*). Huelva: Almonte. Reserva Biológica de Doñana. Laguna de las Pajas, 25 May 1974, *B. Cabezudo* (SEV, as *E. macropoda*). **Turkey**: C5 Adna, Karatas’in 8–10 km kuzeyinde Yenisli Gölü. Kis aylarinda olusan gölcüklerin kenarlari, 26 May 1993, *A. Byfield* (H).

**Table 4:** The number of reviewed sheets and the number of sheets where *E. campylosperma* was found, sorted by the original taxon name on the labels.

Taxon name on the label	Number of reviewed sheets	Number of sheets with seeds	Number of *Elatine campylosperma* sheets
*Elatine macropoda*	188	126	4
*Elatine campylosperma* (incl. *hydropiper* var. *pedunculata; hydropiper* f. *campylosperma*)	76	65	27
*Elatine gussonei* (incl. *hydropiper* var. *gussonei*)	6	5	0
*Elatine major*	8	5	0
*Elatine aquatica*	1	1	0
*Elatine hydropiper*	7	2	1
*Elatine fabri*	6	3	0
*Elatine hardyana*	1	1	0

### Habitat preference

According to Moris’ (1837) and Seubert’s (1842) descriptions, *E. campylosperma* appeared probably in ponds and marshes close to the coastline (‘*in udis maritimis*’). Cultivated plants of Glück (1911) also originated from a maritime site (‘*Sardinien. Golfo Aranci.*’). The known localities of *E. campylosperma* documented in herbaria are also situated close to the coastline (<15 km).

The herbarium sheet from Algeria provides no information about the type of habitat, nor the exact locality, but contains the name of the closest settlement, Annaba. Only a few suitable habitats can be found in the vicinity of this city. According to Samraoui & Samraoui (2008) these sites are likely to be marshes, salt marshes or tidal wetlands, characterized by *Scirpus maritimus*, *Typha angustifolia*, *Tamarix gallica*, *Salicornia europaea* and *Juncus acutus.* French specimens were collected near Rochefort. Wetlands in this area are strongly influenced by marine water sources from sea water in paleotimes (Ladouchea & Weng, 2005), therefore the sediment is characterized by high conductivity and high salt concentration. Greek, Spanish and Turkish specimens were collected from lagoons and temporary marshes.

**Figure 4:**
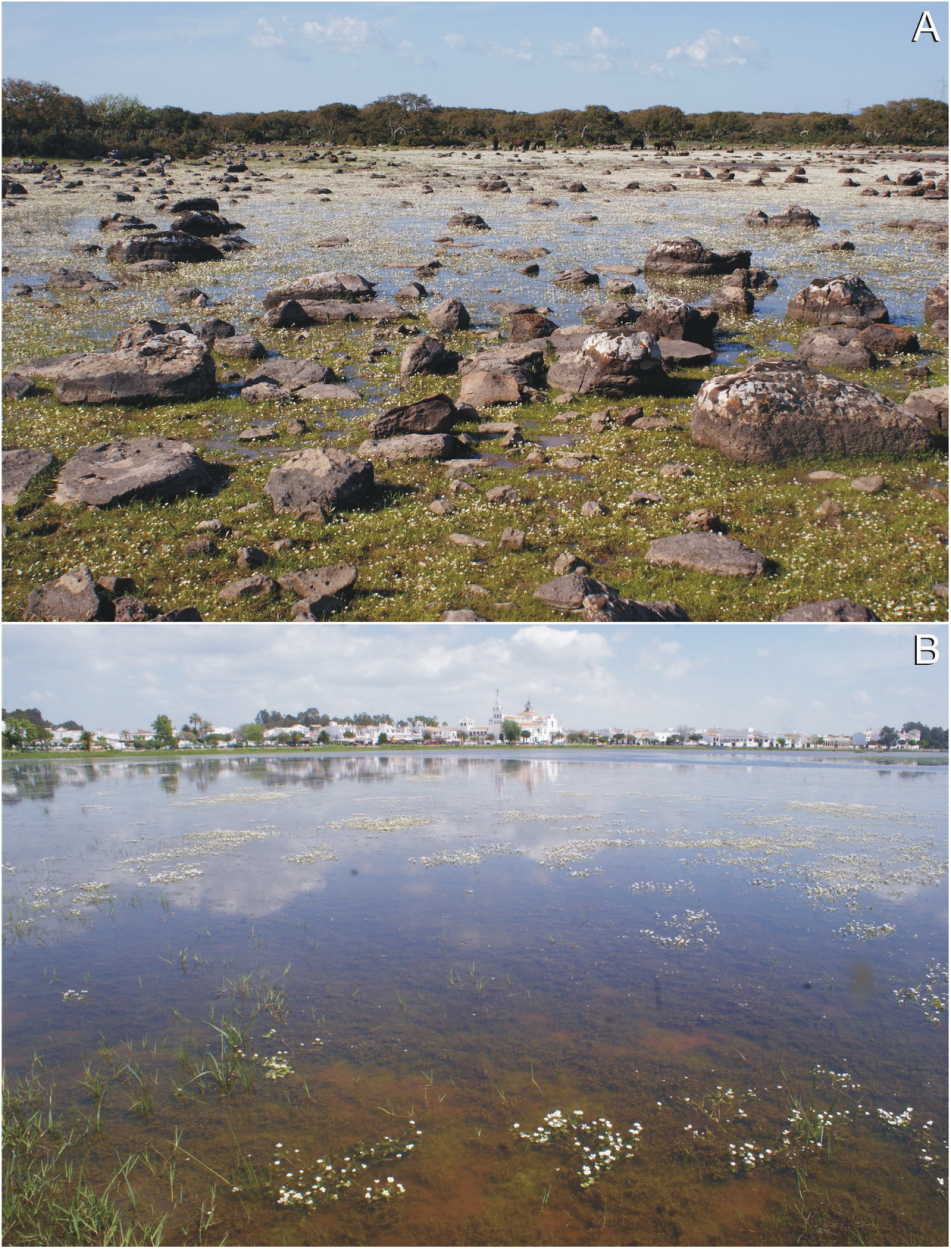
Habitats of *E. campylosperma*. (A) It: Giara di Gesturi; (B) Sp: El Rocío. Photographs: *A. Molnár V*.

In Giara di Gesturi (Italy, Sardinia; Fig. 4A) *E. campylosperma* was found in a Mediterranean temporary pool on basalt substrate at 580 m a.s.l. The water in the pool had low conductivity (378 μS cm^−1^, with pH = 7.8, on 27 April 2012) which is below that is expected in habitats influenced by salt. Accompanying species were *Batrachium aquatile* s.l., *Isoëtes* sp., *Illecebrum verticillatum* L., *Baldellia ranunculoides* (L.) Parl. and *Apium nodiflorum* (L.) Lag. In El Rocío (Spain, Doñana; Fig. 4B) the plant was found in the temporarily and shallowly inundated shoreline of an extensive marsh at 1 m a.s.l. The water in the marsh also had low conductivity (586 μS cm^−1^, with pH = 8.2, on 26 April 2016). Thus, we assume that a balanced climate is more relevant for *E. campylosperma* than a high salinity.

**Figure 5:**
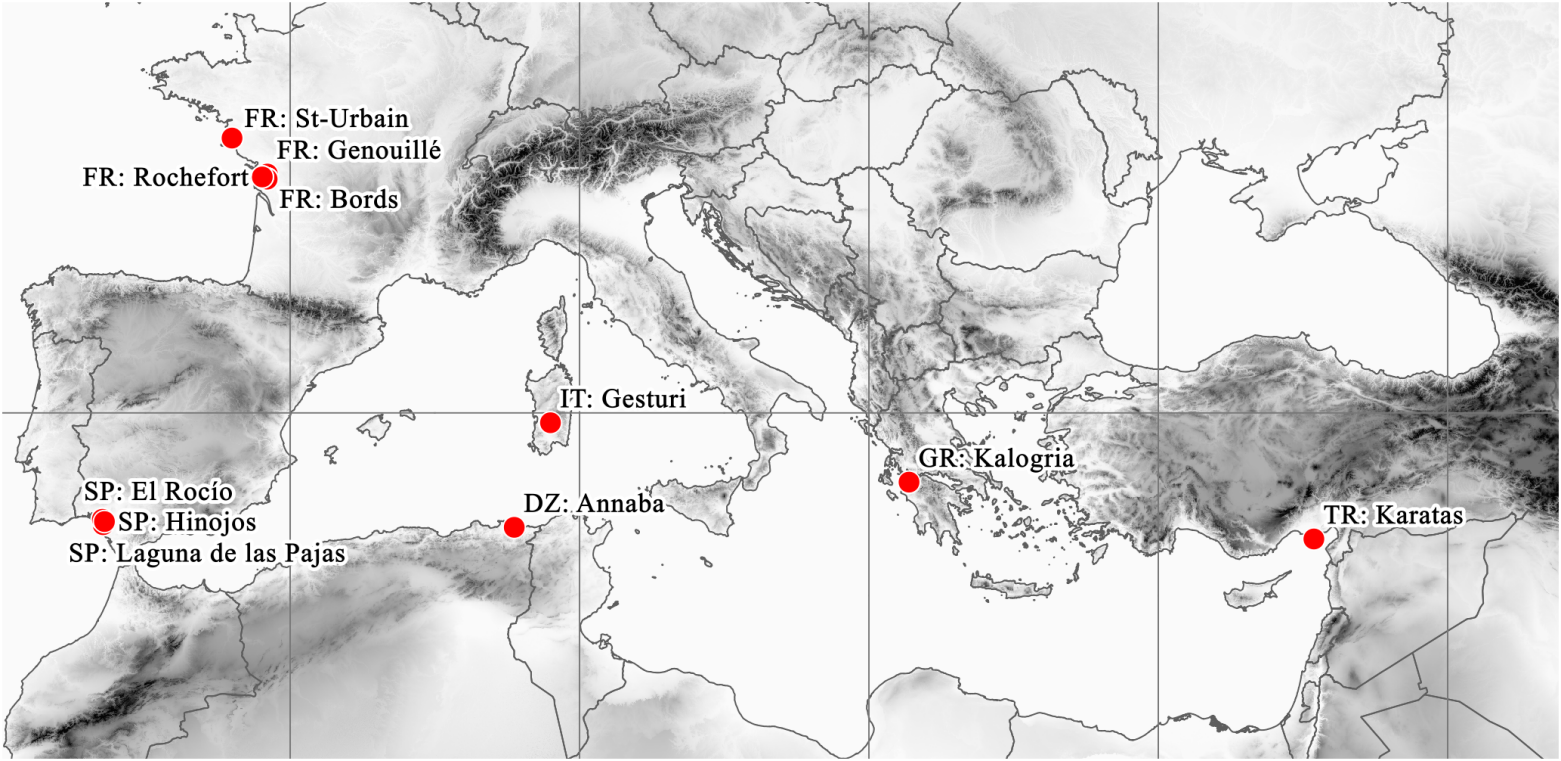
Known distribution area of *E. campylosperma*.

These remote localities of the plant are situated around the Mediterranean basin and the South Atlantic coast of Europe (Fig. 5). Beside the currently confirmed Italian (2012) and Spanish (2013) records, the Turkish (1993) and Greek (1995) records should also be considered as current occurrences. However, there are no confirmed records from France since the second half of the 1800s. Similarly, although the Algerian record is undated, it certainly originates from the early 1800s, given the lifetime of the collector (Adolph Steinheil, 1810–1839).

### Dispersal

We found a total of 77 seeds of *E. campylosperma* in the faecal samples of the Eurasian Coot, with at least one seed in 14 of the 41 samples. Most of the seeds were visibly immature, but eight seeds were found in a ripe stage. Additionally, we found two achenes of *Eleocharis palustris* (L.) Roem. & Schult. and two seeds of unidentified taxa. In the initial one-month germination trial, no seeds germinated. Between the first and the second viability tests during the storage period one *Elatine* seed germinated. During the second germination experiment, a second *Elatine* seed and one of *E. palustris* germinated.

During the field collection of faecal samples we observed more than 200 Eurasian Coot feeding on the carpet-like mats of *E. campylosperma. Elatine* species were already found to be part of the diet of waterbirds (Molodovsky, 1971; Green et al., 2016a). In the natural Doñana marshes (including El Rocío), the coot population shows the highest density (more than 10,000 individuals) around spring time (Rendón et al., 2008), when the waterworts are in rapid development. Only a few studies have addressed the dispersal ability of *Elatine* species. Over short distances, the most important vector is likely to be water (Hayashi et al., 2012), but recent studies showed that waterbirds can act as vectors by endozoochory for wide range of plants (van Leeuwen et al., 2017; Lovas-Kiss et al., 2018a, 2018b). Takács et al. (2017) showed that *Elatine gussonei* seeds can be dispersed by greylag geese *Anser anser* in Doñana. Our results suggest a strong potential for seed dispersal by waterbirds, which provide dispersal over long distances (Green et al., 2016a). This can explain the wide distribution of *E. campyloperma* in the Mediterranean region. The Doñana wetlands are particularly important for migratory waterbirds and have a diverse flora (Green et al., 2016b). Our own surveys show that, besides *E. alsinastrum*, *E. brochonii*, *E. hexandra* and *E. macropoda* (Valdés et al., 2007), this site currently also contains *E. gussonei* (Takács et al., 2017) and *E. campylosperma*.

## Conclusion

By screening the relevant literature, we have detected possible causes for the current ignorance of *E. campylosperma* as a species on its own right. It seems that general recognition of this taxon may have been blurred by both existing unresolved names and, most of all, the long-existing underrating of the taxonomic significance of seed shape in *Elatine* taxonomy, which led to confusion between Mediterranean waterwort taxa in Italian literature, even in ancient sources.

*Elatine campylosperma* is a well-defined species of sect. *Elatinella* subsection *Macropodae*, with a distribution area confined to the Mediterranean zone, where it prefers temporary pools and a balanced climate. Morphologically it is characterized by long flower pedicels and strongly curved seeds with a coat reticulation composed of narrow rectangular pits in the middle row. Further research should be addressed to resolve the taxonomy of the much later described *E. gussonei*, which displays a considerable morphological variability, and clarify its relationship with *E. campylosperma*. The endozoochorous dispersal by waterbirds may account for the wide, though sporadic distribution of *E. campylosperma*.

## Supplemental Information

10.7717/peerj.4913/supp-1 Supplemental Information 1 Raw data of the measured porphological traits.

## Additional Information and Declarations

### Field Study Permissions

The following information was supplied relating to field study approvals (i.e. approving body and any reference numbers): Plants were collected from Doñana under permit 2015107300000771/FQH/MDCG/mes, water chemistry was measured under permit 2014/30, bird faeces were collected under permit 2014/31 from the Doñana Natural Space.

### Data Availability

The following information was supplied regarding data availability: The raw data are provided in a Supplemental File.
